# Biological Characteristics and Genetic Heterogeneity between Carcinoma-Associated Fibroblasts and Their Paired Normal Fibroblasts in Human Breast Cancer

**DOI:** 10.1371/journal.pone.0060321

**Published:** 2013-04-05

**Authors:** Qiongle Peng, Liuyang Zhao, Yixuan Hou, Yan Sun, Liyang Wang, Haojun Luo, Huimin Peng, Manran Liu

**Affiliations:** 1 Key Laboratory of Laboratory Medical Diagnostics, Chinese Ministry of Education, Chongqing Medical University, Chongqing, China; 2 Chongqing Key Laboratory of Laboratory Medical Diagnostics, Chongqing Medical University, Chongqing, China; 3 Department of Cell biology and Genetics, School of Basic Medicine Science, Chongqing Medical University, Chongqing, China; 4 Experimental Teaching Center, Chongqing Medical University, Chongqing, China; 5 Department of Endocrine and Breast Surgery, The First Affiliated Hospital, Chongqing Medical University, Chongqing, China; B.C. Cancer Agency, Canada

## Abstract

**Background:**

The extensional signals in cross-talk between stromal cells and tumor cells generated from extracellular matrix molecules, soluble factor, and cell-cell adhesion complexes cooperate at the extra- and intracellular level in the tumor microenvironment. CAFs are the primary type of stromal cells in the tumor microenvironment and play a pivotal role in tumorigenesis and development. Hitherto, there is hardly any systematic analysis of the intrinsic relationship between CAFs function and its abnormal signaling pathway. The extreme complexity of CAFs’ features and their role in tumor development are needed to be further investigated.

**Methodology/Principal Findings:**

We primary cultured CAFs and NFs from early stages of breast cancer tissue and identified them using their biomarker by immunohistochemistry for Fibronectin, α-SMA and FAP. Microarray was applied to analyze gene expression profiles of human breast CAFs and the paired NFs. The Up-regulated genes classified by Gene Ontology, signal pathways enriched by DAVID pathway analysis. Abnormal signaling pathways in breast cancer CAFs are involved in cell cycle, cell adhesion, signal transduction and protein transport being reported in CAFs derived from other tumors. Significantly, the altered ATM signaling pathway, a set of cell cycle regulated signaling, and immune associated signaling are identified to be changed in CAFs.

**Conclusions/Significance:**

CAFs have the vigorous ability of proliferation and potential of invasion and migration comparing with NFs. CAFs could promote breast cancer cell invasion under co-culture conditions through up-regulated CCL18 and CXCL12. Consistently with its biologic behavior, the gene expression profiling analyzed by microarray shows that some of key signaling pathways, such as cell cycle, cell adhesion, and secreting factors play an important role in CAFs. The altered ATM signaling pathway is abnormally active in the early stage of breast cancer. The set of immune associated signaling may be involved in tumor cell immune evasion.

## Introduction

Accumulating evidences show that the progression of malignant tumors does not depend exclusively on the cancer cells themselves, but is also deeply influenced by the tumor microenvironment [1]. Tumor microenvironment is a whole system which includes tumor cells, stroma cells (such as, adipocytes, fibroblasts, endothelial cells, infiltrating immune cells) and extracellular matrix (ECM) [2], [3]. Carcinoma-associated fibroblasts (CAFs), the activated fibroblasts, which were named by Tlsty’s group in the tissue of prostatic cancer in 1999, are the primary type of host cells in the tumor microenvironment [4]. CAFs were found in almost all solid tumor tissue (e.g. cancers of the colon, lung, liver, prostate, pancreas and gastric cancer), and play an assignable role in tumor development by cell-cell interaction or cross-talk with tumor cells through secreting growth factors, cytokines and chemokines [2], [4]–[6]. Therefore, CAFs are thought to be “the dark side of coin” in tumor development [1].

In contrast to normal fibroblasts, CAFs have some of phenotypical and functional abnormality. These alterations of CAFs may be due to its stable gene expression changes. So, whole breast carcinomas stromal by microdissection were explored to analyze the gene expression profiling [7]. These studies have led to new classifications and risk stratification of breast carcinomas into several molecular subtypes based on their gene expression signatures [8], [9]. The global gene expression in the stromal cells has also been shown to powerfully predict prognosis and treatment response [10], [11]. A variety of differences have been identified between breast carcinoma-associated stroma and its paired normal mammary stroma, primarily resulting in increased expression of cytokines (EGF, HGF PDGF, TLL-12, SSP-1, POSTN, CXCL-12, and CXCL14), extracellular matrix (ECM) molecules (FBN1, FB2M, SPARC, ADRA2A and ADM) and proteases (MMP-1, MMP-2, MMP-13) [10]–[16]. These factors were involved in cross-talk between stromal cells and tumor cells by directly or indirectly pattern to promote tumor cell proliferation, cell adhesion and invasion, ECM remodeling [17]. Although, these studies give us the overall configuration of the tumor microenvironment, there are still poor on the role of each member in the tumor stroma, like CAFs, to contribute to the tumor development.

Until most recently, the distinguishable features and gene expression profile of CAFs and NFs have been described in breast cancer by Bauer et al. [18]. Their studies show that up-regulated WISP1, collagen type-X (COL10A) and TGF-β isoforms in CAFs activate paracrine Wnt-1 signaling in human breast cancer cells or lead to abnormal ECM production in stroma separately. Loss of expression of AKR1C1 and AKR1C2 may alter the hormonal milieu in breast carcinomas, and down-regulation of KLF4 participates in the differentiation of resting fibroblasts to myofibroblasts. However, there is still no systematic analysis of the intrinsic relationship between breast cancer CAFs function and its abnormal signaling pathway. The extreme complexity of breast cancer CAFs feature and its roles to tumor development are needed to be further investigated. Furthermore, whether these previous finding in CAFs derived from western country women are also existed in patients of other races.

In light of these facts, the principal purpose of this work is to investigate the possible innate nature of biological and genetic heterogeneity between human breast CAFs versus matched NFs in Chinese women. We identified 824 differentially expressed genes: 809 of these were up-regulated, and 15 were down-regulated in CAFs, which included 8 cytokines. The abnormality of CAFs genes and its associated signaling pathways could be involved in cell cycle, cell adhesion, signal transduction and protein transport, which were proved in CAFs of other tumor microenvironment [19]–[21]. Interestingly, we firstly discovered that the altered ATM signaling pathway, which was recently found related to reactive oxidative stress (ROS) [22], [23], is abnormally activation in the early stage of breast cancer. Cell cycle regulated signaling, such as P53 signaling pathway, RB tumor suppressor/checkpoint signaling, regulation of cell cycle progression by Plk3, Cdc25 and Chk1 regulatory pathway are identified to be changed in CAFs. A set of immune associated signaling are abnormality in CAFs, which include natural killer cell mediated cytotoxicity, Fc gamma R-mediated phagocytosis, antigen processing and presentation, immune network for IgA production, Lck and Fyn tyrosine kinases in initiation of TCR Activation, B lymphocyte cell surface molecules. And more importantly, we found that CAFs would be able to promote breast cancer cell invasion under co-culture condition through up-regulated CCL18.

## Materials and Methods

### Tissue Samples

All the human breast tumor tissues and its adjacent normal mammary tissues were collected at the time of surgical reaction at the First Affiliated Hospital of Chongqing Medical University. None of the patients received adjuvant therapy before surgery. Specimens used in this study were approved by the Ethics committees of Chongqing Medical University.

### Isolation and Culture of Primary Fibroblasts

Tumor tissue and paired normal mammary tissues were washed 3 times with sterile PBS with antibiotics (100 U/ml penicillin, 100 µg/ml streptomycin and 50 µg/ml gentamycin). The tissues were minced with sterile scissors in a fresh sterile culture dish. After digestion with 0.1% collagenase type I (C0130, Sigma, Saint Louis, MO) at 37°C for 8–12 h [24], tissues were carefully pipetted up and down for a couple of times using culture medium. The mixtures were centrifuged and washed with DMEM to remove the fat and tissue debris. Then, the mammary tissues were cultured in DMEM with 10% fetal bovine serum (FBS, Gibco, Australia) for about two days. Removed the suspending cells or tissue, the most adherent cells were fibroblasts. The primary fibroblasts isolated from tumor tissues were named “CAFs”, and from tumor paired normal tissues named “NFs”. Cell purity was identified by immunohistochemistry for Fibronection, α-SMA and FAP.

### Immunofluorescence

Immunofluorescence staining for Fibronection, α-SMA and FAP following the standard described process previously [25] and manufacture’s protocols. Briefly, cells were grown on cover slips for 24 hours and fixed within 4% paraformaldehyde, treated with 0.1% triton-100 and blocked with 5% goat serum. Then, cells were incubated with primary antibodies (1∶150) for targeting fibronectin (F3648, Sigma, St. Louis, MO), α-SMA (ab5694, Abcam Cambridge) and FAP (ab53066, Abcam Cambridge, Neomarkers, Fremont, CA) at 4°C for overnight. After washing with PBS, cells were then stained with a FITC-labeled goat anti-rabbit secondary antibody (1∶100, ZF-0311, Zhongshan Goldenbrdge Biotechnology, Beijing, China) and DAPI. Immunofluorescent images were taken using a Nikon Eclipse 80 i microscope (Eclipse 80 i, Tokyo, Japan; magnification×100).

### MTT and Flow Cytometric Analysis

Cell growth was determined by 3-(4,5-dimethylthiazol-2yl)-2,5-diphenyl tetrazoliumbromide (MTT) assay. Cells were plated into 96-well plate at 5×10^3^ cells per well in 200 µl of complete growth medium. Cell growth was measured every day. Incubating for the designed time, MTT (5 mg/ml) was added to each well and incubated for 4 h. After careful removal of the medium, 0.1 ml of isopropanol with 0.04 N of HCl was added to each well, and the plates were shaken on a rotator for 20 min at room temperature. The absorbance was recorded on an ultraviolet spectrophotometric reader at a wavelength of 570 nm. The independent experiments were repeated for five times.

S-phase was analyzed by using laser scanning cytometry. Cells were processed by standard methods using propidium iodide staining of cell DNA as previously described [26]. A minimum of 20,000 events was collected to maximize the statistical validity of the compartmental analysis. Triplicate independent experiments were done.

### Preparation of Conditioned Medium

MDA-MB-231 cells, CAFs and NFs were cultured in complete growth DMEM for 48 h to approximately 80% confluence. The medium was changed to 0.5% FBS DMEM and cells were kept in culture for another 30 to 36 h. For neutralization experiments, neutralizing antibodies against human CXCL-12 (500 ng/ml), CCL18 (10 µg/ml), CCL4 (1.5 µg/ml) and control IgG (R & D Systems, Minneapolis, MN) were separately preincubated at 37°C with supernatant for 1 h before performing migration assays. The medium was centrifuged at 1,200×g for 15 min, and the supernatant was collected as conditioned medium (CM).

### Cell Migration and Invasion Assay

Cell invasion or migration assay was measured via modified Boyden chamber assay as described previously [25]. Briefly, 2×10^4^ MDA-MB-231 cells in 200 µl serum-free medium were seeded in the wells of 8 µm-pore membrance modified Boyden chambers (Millipore, Darmstadt, Germany) coated with ECM (1∶7.5) (Sigma, St. Louis, MO). 10% FBS Medium, CM, CM with secreting factor CXCL-12 (100 nM), CCL18 (500 ng/ml) or CCL4 (100 nM) was separately added into the lower or upper chamber as the designed purpose. After 6 h or 12 h of incubation at 37°C and CO_2_ at 5%, cells adhered to the upper surface of the filter were removed using a cotton applicator. Stained with hematoxylin in methanol, the invading cells on the opposite side of the filter were counted. The data represent at least three experiments done in triplicate (mean±standard error).

### RNA Isolation, Quantitative Real-time PCR and Microarray Analysis

Total RNA isolated from six paired CAFs and NFs using TRIzol (Invitrogen, Carlsbad, California, USA) according to the manufacturer’s protocol. RNA quantity was determined by agarose gel electrophoresis and by spectrophotometry. Probe synthesis and hybridization performed as the manufacture’s instruction were used to probe the Agilent Human Whole Genome Oligonucleotide Microarray (44 K; Agilent, Santa Clara, CA, USA). Data were generated after scanning by using an Agilent Scanner (Agilent, Santa Clara, CA, USA) and extracted using Agilent Feature Extraction Software v9.5 (Agilent, Santa Clara, CA, USA). The raw data were normalized using quantize normalization and then analyzed by GeneSpring GX v10.0 (Agilent, Santa Clara, CA, USA). Paired Significance Analysis of Microarray (SAM) was applied to identify differentially expressed mRNA in CAFs and NFs. SAM output was filtered by q = 0.1. Heatmap.2 function (R v2.14.0, gplots package v2.9.0) was used to plot the scaled expression data of differentially expressed mRNAs.

An additional two expression datasets (GSE20086 [18] and GSE29270) on breast cancer associated fibroblasts were downloaded from the GEO website (http://www.ncbi.nlm.nih.gov/geo/). The raw data of six matched CAFs and NFs in GSE20086 were normalized using the R package gcRMA [27], GCRMA normalized. The author’s processed data of 15 matched CAFs and NFs in GSE29270 were used in next analysis. Paired SAM were also used to analysis the different expression genes in CAFs versus NFs. In cytokines analysis process, the genes at least fold change >1.5 with an estimated FDR<10% were considered as dysregulated genes.

RNA was subjected to reverse transcription reactions by using the PrimeScript RT reagent Kit (Takara, Dalian, China). To confirm the differential gene expression pattern revealed by microarray analysis, 9 of randomly selected genes (CDC6, FLI1, S100A9, CDK1, PLK1, MMP9, PECAM1, SHC2 and C9ORF135) were re-proved by qRT-PCR assay. Specific Real-time PCR primers for each of selected genes were listed as Table 1. qRT-PCR was performed by a Bio-Rad CFX Manager instrument (Applied Biosystems) at 94°C for 15 Sec, Tm°C for 20 Sec and at 65°C for 30 Sec using SYBR Premix Ex Taq TM II (Takara, Dalian, China). GAPDH were used as internal control for normalizing different samples.

**Table 1:** Primer sequences for qRT-PCR.

Gene	Forward 5‘–3'	Reverse 5‘–3'	Size (bp)
FN	AACTTCCTGGTGCGTTACTCA	TGTGCTCTCATGTTGTTCGT	156
α-SMA	GAGGCACCCCTGAACCCCAA	ATCTCCAGAGTCCAGCACGA	153
FAP	TGTTCCAGCAATGATAGCC	CTGCTTTCTTCTATATGCTCC	186
CDC6	CCAGGCACAGGCTACAATCAGT	ACACGAGGAGAACAGGTTACGG	126
S100A9	GCACCCAGACACCCTGAACC	ACCCTCGTGCATCTTCTCGT	211
FLI1	CCACCCTCTACAACACGGAA	ATGTTATTGCCCCAAGCTC	166
CDK1	GGATCTACCATACCCATTGAC	TGGCTACCACTTGACCTGT	120
PLK1	AATTACATAGCTCCCGAGGTG	AGCCAGAAGTAAAGAACTCGTC	118
MMP9	TCCCTGGAGACCTGAGAACC	GGCAAGTCTTCCGAGTAGTTT	307
PECAM1	ACTCAAATGATCCTGCGGTA	ACTTAACATTTTGGCATGGGA	98
SHC2	GCACCTGTATGTCAACACCCA	TCCTCAAAGGGTCGCATGTCA	90
C9ORF135	TCCCATGGATAGCCTTGACA	GTGCCAGGAATACACCAAC	160
CXCL12	CAGAGCCAACGTCAAGCATC	ATCCACTTTAGCTTCGGGTC	117
CCL18	CCGCCTCGTCTATACCTCC	CACTTCTTATTGGGGTCAGC	141
CCL4	TTCCTCGCAACTTTGTGGTA	CAGGTCATACACGTACTCC	140
CXCL12	CAGAGCCAACGTCAAGCATC	ATCCACTTTAGCTTCGGGTC	117

**Note:** FN, Fibronectin; α-SMA, alpha-smooth muscle actin; FAP, fibroblast activation protein; CDC6, cell division cycle 6 homolog; S100A9, S100 calcium binding protein A9; FLI1, Friend leukemia virus integration 1; CDK1, cyclin-dependent kinase 1; PLK1, polo-like kinase 1; MMP9, matrix metallopeptidase 9; PECAM1, platelet/endothelial cell adhesion molecule; SHC2, SHC transforming protein 2; C9ORF135, chromosome 9 open reading frame 135; CXCL12, chemokine ligand 12 (stromal cell-derived factor 1);CCL18, chemokine (C-C motif) ligand 18; CCL4, chemokine (C-C motif) ligand 4.

### ELISA Assay

Supernatant from NFs or CAFs were harvested and subjected to the enzyme-linked immunosorbent assay (ELISA) kit for measuring the immunoreactive levels of CXCL-12, CCL18 and CCL4 (R & D Systems, Minneapolis, MN) according to the instructions as supplied by the manufacturer. The ELISA assay was carried out in duplicate in three separate experiments.

### Gene Ontology and Pathway Analysis

For detection of significantly over-represented GO biological processes, the Onto-Express analysis tool [28] was used and the permutated P-value cut-off was set below 0.05. The pathway analysis of differentially expressed mRNAs was conducted through the DAVID functional annotation clustering tool [29] and the significantly changed signaling pathways were selected based on P<0.01.

### Statistical Analysis

Statistical analysis was done using SPSS standard version 13.0 software. Data was shown as means ± SD from at least three independent determinations. Significance of difference was analyzed using two-tailed Student’s t tests. A p value of less than 0.05 was considered significant differences.

## Results

### Isolation and Identification of CAFs and NFs

**Figure 1:**
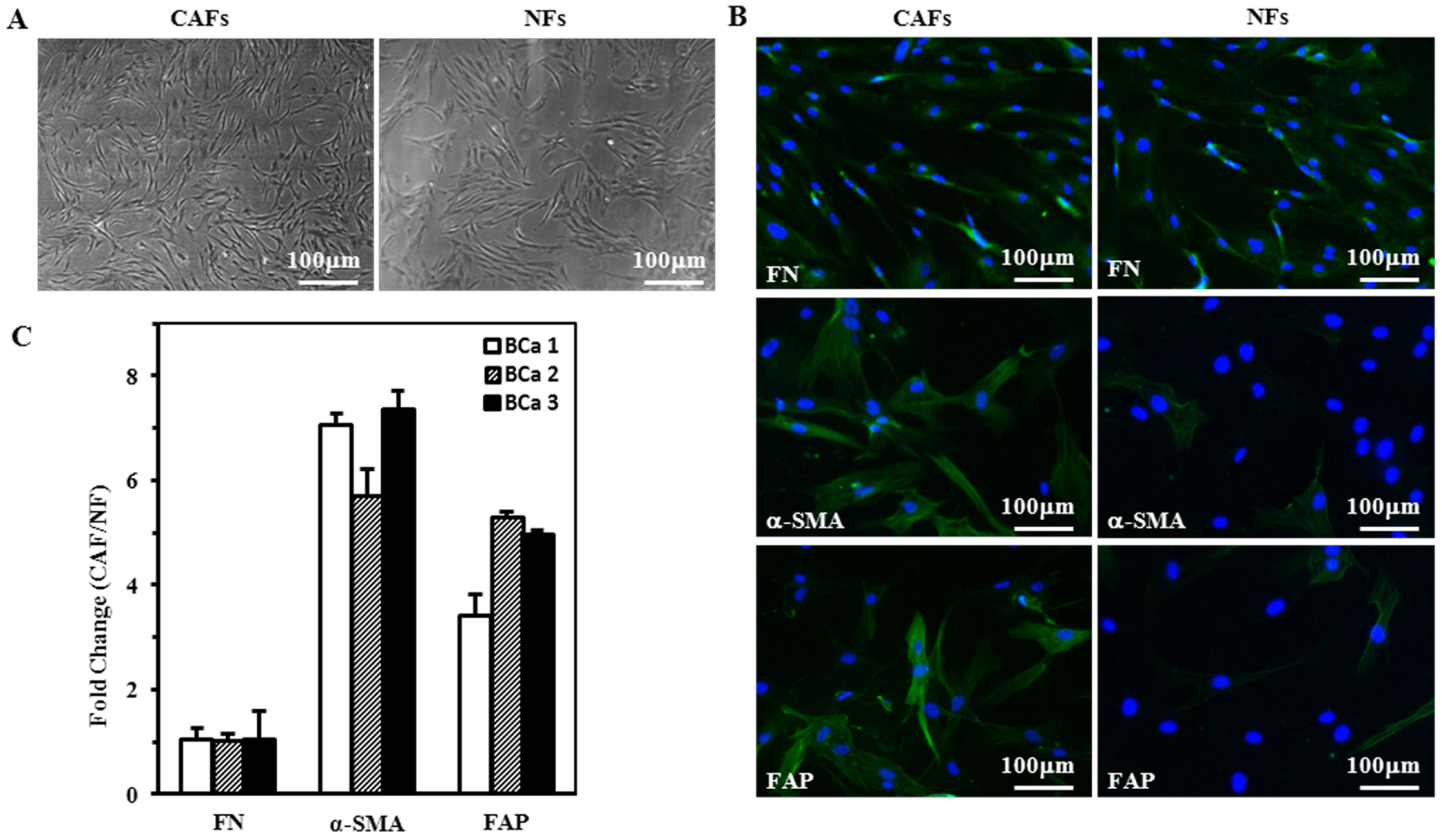
Characterization of fibroblasts isolated from human breast tissue samples. A. Representative cell morphology of CAFs and NFs. B. Identification of CAFs using fibroblast biomarker fibronectin, and CAFs specific biomarker α-SMA and FAP by immunofluorescence staining. C. The biomarker gene expression in CAFs and NFs was re-proved by qTR-PCR, and its relative fold change in CAFs and NFs was displayed. The CAFs and NFs are positive for fibroblast biomarker fibronectin, CAFs specific biomarker α-SMA and FAP are high expression in CAFs (magnification 100× (cell morphology); 200× (Biomarker immunofluorescence staining).

CAFs and their paired NFs were successfully isolated from six primary breast carcinomas and from adjacent normal breast tissue. The primary fibroblasts were successfully cultured in DMEM with 10% FBS. Both CAFs and NFs could be grown for at least 10 passages. Both CAFs and NFs showed spindle-like morphology (Fig. 1A). But the senescence-like cell would be appeared when the cell culture was more than 6 passages (Data not shown).

The purity of CAFs was identified by fibronectin, which is the fibroblast biomarkers, and CAFs-specific biomarkers including α-SMA and FAP, respectively. NF and CAFs were positive for fibronectin, more than 95% of CAFs were stained by α-SMA and FAP (Fig. 1B). These biomarker expression changes were reproved by qRT-PCR in 3 paired of CAFs and NF (Fig. 1C). These data demonstrated that the purified CAFs were isolated from tumor tissue.

### The Noticeable Heterogeneity of Proliferation, Migration and Invasion between CAFs and NFs

**Figure 2:**
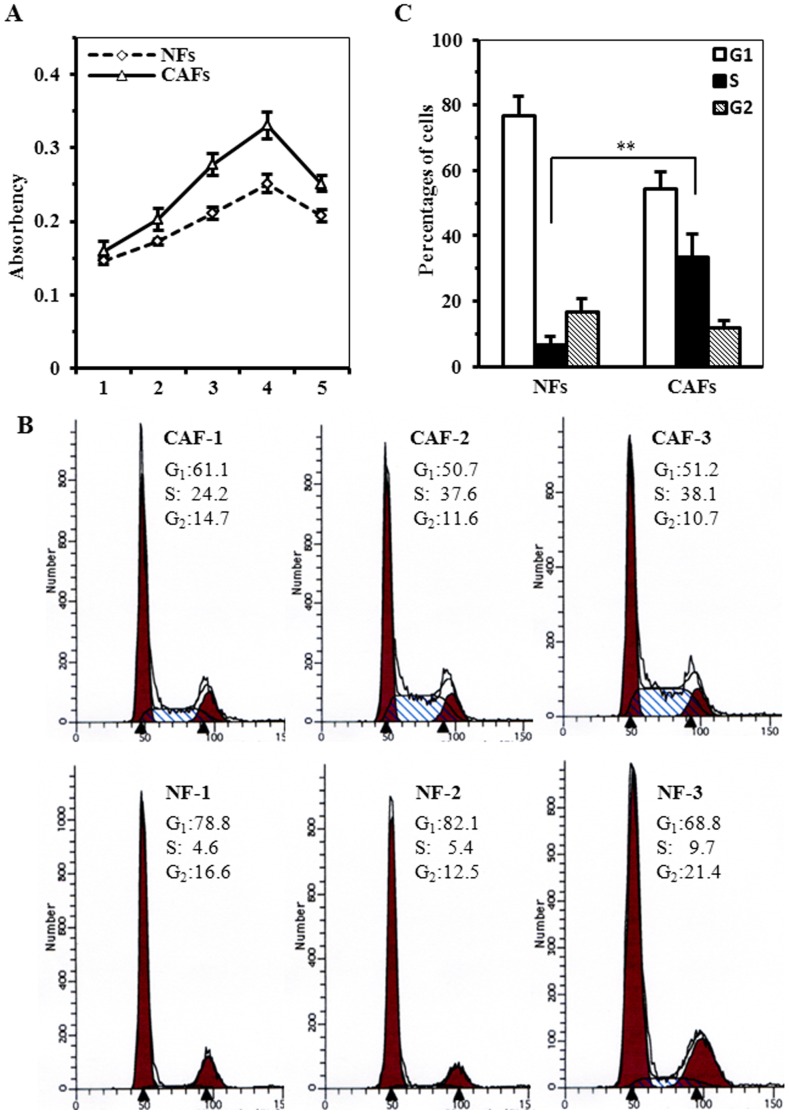
Significant difference of proliferation between CAFs and NFs. A. Cell proliferation determined by MTT assay for three of CAFs and NFs. B. Representative DNA content of CAFs and NFs were tested by flow cytometry. C. The percentages of cells in each of cell cycle phases shown by histogram for CAFs vs. NFs. The data were shown as mean±SD for N≥3 separate experiments (**P<0.01).

**Figure 3:**
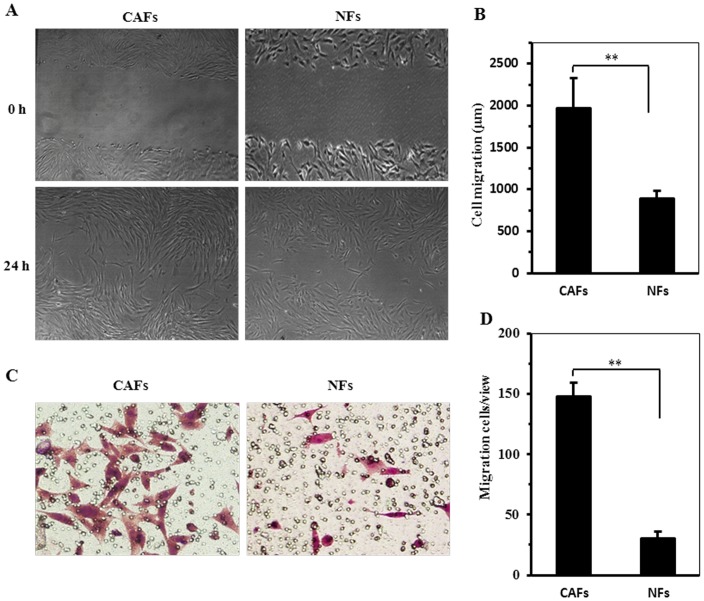
The migration and invasion of CAFs compared with NFs. A. Cell migration capability was determined by wound healing assay. B. The distance of wound closure (compared with control at 0 h) was measured in four-independent wound sites each group after 24 h. C. The invasion capability of CAFs and NFs was determined by Transwell assay. D. The migrated cells of CAFs and NFs shown by a histogram. Data were shown as mean±SD of 5 repeats. (**P<0.01), (magnification A. 100×; C. 200×).

The cell growth assay revealed that CAFs were endowed a significantly growth rate than that of NFs in the same culture conditions (P<0.05; Fig. 2A). The percentage of cells in the S phases measured by flow cytometry was significantly higher in CAFs (33.3±7.1, N>3) compared with NFs (6.6±2.5, N>3) (P<0.05). These data suggested that CAFs had a stronger capacity for proliferation than their paired NFs (Fig. 2B–C). Similarly, CAFs had strong potent ability of migration (Fig. 3A–B) and invasion (Fig. 3C) than its paired NFs.

### CAFs Stimulate Tumor Cell Growth, Migration and Invasion

**Figure 4:**
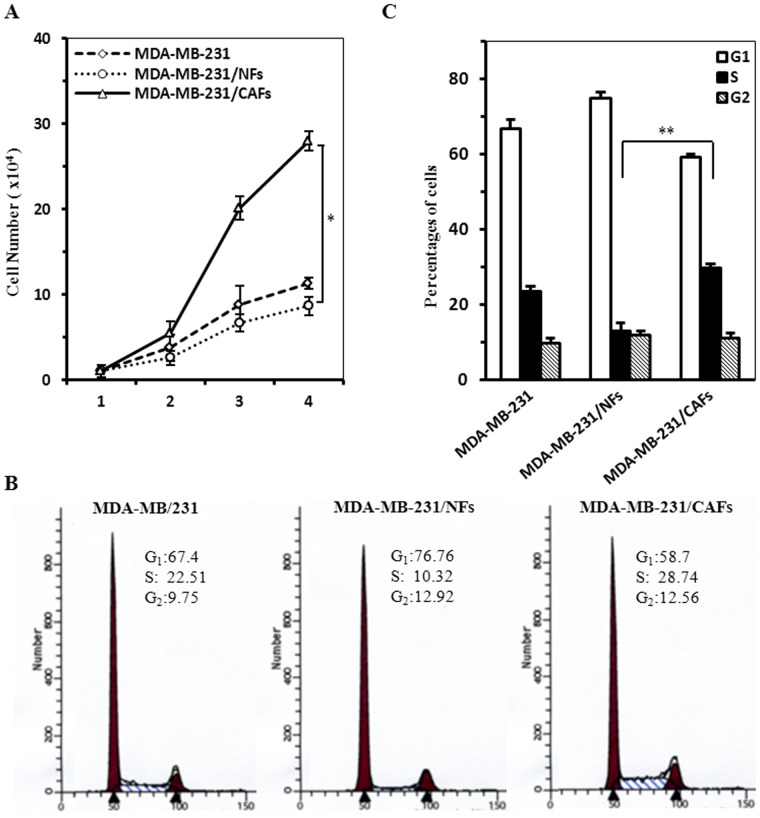
CAFs promote proliferation of MDA-MB-231 cells in co-culture system compared with NFs. A. Cell growth curve determined by cell count. B. Representative DNA content of MDA-MB-231 cultured with normal medium and with conditioned medium derived from CAFs and NFs determined by flow cytometry. C. The percentages of cells in each of cell cycle phases shown by histogram for MDA-MB-231. The data were shown as mean±SD for 3 separate experiments (*P<0.05; **P<0.01).

**Figure 5:**
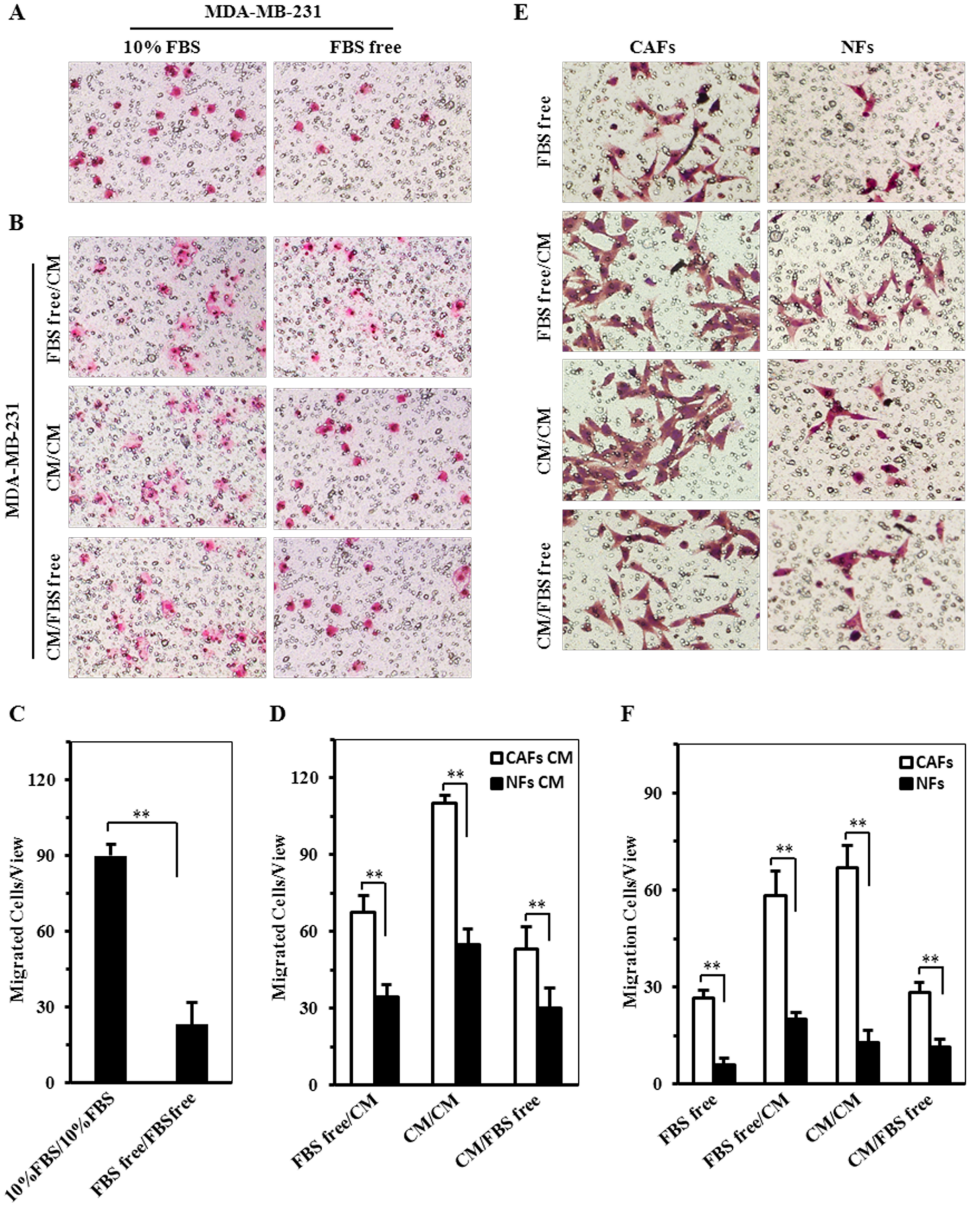
The potential interaction of CAFs and tumor cells on cell invasion. A, C. MDA-MB-231 invasion ability was tested by Transwell assay cultured in the medium with FBS or FBS free (A), and migrated cells were shown by histogram (C). B, D. The migrated MDA-MB-231 cells were checked by Transwell assay (B) in the co-culture system using condition medium derived from CAFs and NFs. And (D) the migrated cells shown by histogram. E, F. The invasive potential of CAFs and NFs were determined by Transwell assay (E) in the co-culture system using condition medium derived from MDA-MB-231 cells. The histogram to show the average migrated cells each view (F). Data is representative of 5 views (**P<0.01), (magnification: A, B 100×; E 200×).

In order to understand the interaction cross-talk of CAFs with tumor cells in the tumor microenvironment, co-culture system was employed to display their role in the cell proliferation and invasion. In contrast to MDA-MB-231 cultured in CM derived from NFs (NFs CM) and control, MDA-MB-231 cultured in CM derived from CAFs (CAFs CM) had significant proliferation (P<0.05; Fig. 4A) assayed by cell growth curve. The proportion of MDA-MB-231 cells in the DNA synthesis (S) phase cultured in CAFs CM (29.8±0.8, N = 3) was obviously more than that in NFs CM (13.0±2.2, N = 3; P<0.01) and control (23.5±1.4, N = 3; P<0.05) (Fig. 4B–C). Collectively these studies demonstrate that CAFs had a stronger capacity promoting tumor cell proliferation than their paired NFs in the breast tumor microenvironment. Additionally, the increasing cell invasion was found in the co-culture system of MDA-MB-231 cells with CAFs CM, as compared with the system containing NFs CM or control medium (Fig. 5A–D).

To examine whether tumor cells play a role in the biologic behavior of fibroblasts in the stroma, the effect of MDA-MB-231 on the migration of CAFs and NFs was compared. As predicted, more migrated cells were tested in the co-culture of CAFs and CM from MDA-MB-231 (Fig. 5E–F). These data indicated that different effects existed in the cross-talk of tumor cells and fibroblasts.

### Genes Differentially Expressed between CAFs and NFs

**Figure 6:**
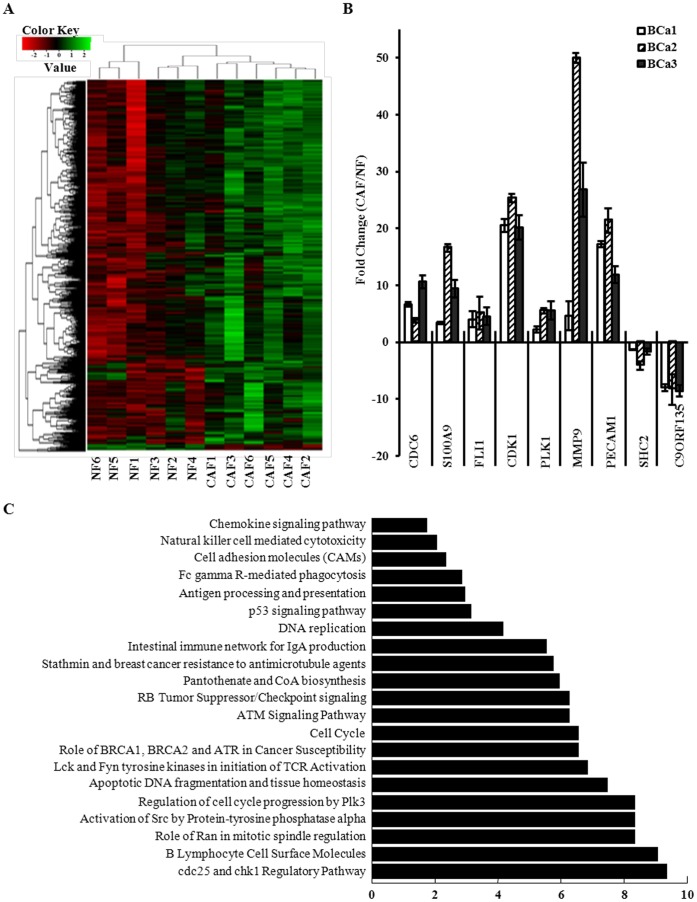
Gene heterogeneity between CAFs and NFs. A. HeatMap of gene expression data obtained by Agilent micro-arrays analysis from CAF and NF isolated from all six cases (BCa 1, 2, 3, 4, 5, 6) were subjected to unsupervised cluster analysis. B. The mRNA levels of 9 genes selected randomly were analyzed by qRT-PCR in CAF and NF from 3 patients with breast cancer. The data were shown as fold change in CAF vs. NF. C. Signaling pathway analysis of enriched processes and signaling pathway in CAFs vs. NFs.

To reveal the essence of the heterogeneity of biologic characteristic between CAFs and NFs in the breast carcinoma microenvironment, gene expression profile was investigated by micro-array assay. Total RNA derived from six paired CAFs and NFs isolated from primary breast infiltrating ductal carcinomas of grade ΙΙ were used in this analysis. Gene expression profiles of pooled CAFs and their pooled normal counterparts were obtained by microarray analysis using the Agilent Human Whole Genome Oligonucleotide Microarray (Fig. 6A). A total of 25,206 probe sets (transcripts) were present in human breast carcinomas-derived fibroblasts relative to their normal controls. Compared with the expression profile of NFs, 809 up-regulated genes (0.9%) and only 15 down-regulated genes (0.45%) were detected in CAFs using an arbitrary cutoff line of signal log ratio of ≥1.8 or ≤−1.8. Chemokine (C-C motif) ligand 18 (CCL18), chemokine (C-C motif) ligand 12 (CXCL12), cell division cycle 6 homolog (CDC6); Friend leukemia virus integration 1 (FLI1); S100 calcium binding protein A9 (S100A9), cyclin-dependent kinase 1 (CDK1), polo-like kinase 1 (PLK1), matrix metallo peptidase 9 (MMP9), and platelet/endothelial cell adhesion molecule (PECAM1) were predominantly over-expressed at high levels in CAFs whereas SHC transforming protein 2 (SHC2) and chromosome 9 open reading frame 135 (C9ORF135) were down-regulated with a high array intensity (Table S1 and Table S2).

The up-regulated genes encoded for secreted proteins were mostly classified into groups of extracellular region, cell proliferation, and signal transducer activity (Table S3). To validate the microarray data, the expression level of 9 randomly selected genes including 7 of up-regulated genes and 2 of down-regulated genes were re-proved by qRT-PCR between CAFs and NFs (Fig. 6B). Compared with NFs, the expression pattern of all differentially expressed genes in CAFs detected by qRT-PCR was consistent with the microarray results.

### The Unanimity Heterogeneity of Biological Characteristics and Genes Expressed in CAFs

Microarray assay displayed that most genes up-regulated in CAFs have oncogenic function, including growth factors (FGF2, VEGFC, PDGFC, CASC5, and HGFAC), transcription regulators (MKX, RUNX3, ATAD2, FOXM1, and PTTG1), cell proliferation-associated genes (CENPF, PLK1, BUB1, KIF15, and CRIP1) and members of the Wnt signal pathway (WNT1, WNT5A, LRP6, TCF4, and GBP4). Several genes encoding secreted proteins, such as CCL18, CXCL12, MCM10, ADAMDEC1, MMP9, and S100A9 were also significantly up-regulated in CAFs (Table S3). All of these secreted proteins are able to induce cell proliferation, implying that CAFs play a role in the proliferation of tumor cells.

Additionally, microarray results suggest that CAFs may facilitate invasion and metastasis of breast tumor cells. The data listing in Table S1 shows that CAFs promote tumor cell invasion mainly via two of mechanisms: (a) altering tumor microenvironment interactions (tumor-stroma and tumor-tumor) by increasing cell adhesion (PECAM1, ITGAX, CCL4, LPP, and SIGLEC8) and cell-cell interaction signaling (CCL18, DLGAP5, C1QA, STAB1, and TEK), and (b) promoting extracellular region degradation (LYZ, C1QB, MMP9, IGLL1, and IGHG1) surrounding tumor cells (Table S3).

Furthermore, Gene Ontology analysis of enriched processes and signaling pathways showed that CAFs enriched many serviceable signaling pathways in contrast to NFs in human breast cancer. Maximum change pathways in CAFs were cdc25 and chk1 regulatory pathways associated with cell cycle. Compared with NFs, the cell cycle and DNA replication signal pathway in CAFs were strong activated for 4 and 6 times, respectively. The alteration of the ATM signaling pathway was absolute significantly. It was recently reported that the ATM signaling was activated under oxidative stress [22]. Our preliminary data indicated that activated ATM signaling was in responsible for cell proliferation of fibroblasts (Data not shown). The change of other pathways was also assignable, such as DNA replication signal pathways, chemokine signaling pathways, PI3k signaling pathways, abnormal activated p53 signaling pathway (Fig. 6C). Herein, the heterogeneity of genes expressed between CAFs and NFs was consistent with their heterogeneity of biological characteristics.

### The Common Cytokines-CCL18 Obtained by Compared with Published Microarray Data

**Figure 7:**
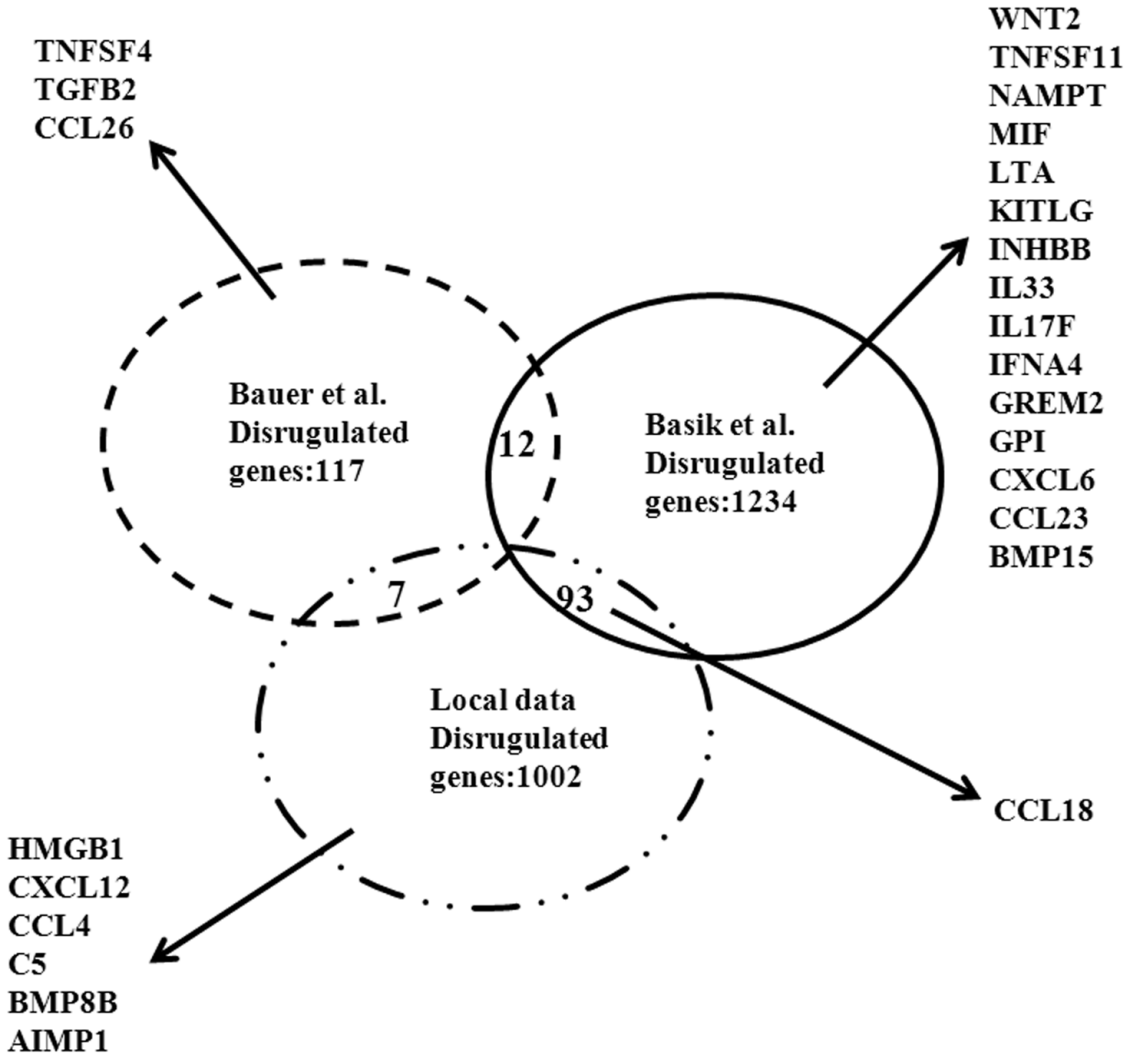
The common cytokines-CCL18 was obtained by compared with published microarray data. The number of dysregulated difference genes and shared genes from current work and two other studies in breast cancer associated CAF versus paired NF was displayed in each group. Dysregulated Cytokines in each study were listed. Only the common cytokines-CCL18 was obtained compared local data with Basik’s microarray data (GSE29270).

To compare the shared genes in current work with previous publication in breast cancer CAFs, the mRNA microarray data of breast cancer CAFs and its paired NFs stored in National Center for Biotechnology Information Gene Expression Omnibus (GEO; accession number GSE20086 [18] and GSE29270) were used. As shown in Fig. 7, the dysregulated genes of CAFs are 136 (M Bauer), 1339 (M Basik), and 1102 in current work, respectively. The shared dysregulated genes among each of these data were 12 (Bauer’s data vs. Basik’s data), 7 (Local data vs. Bauer’s data), and 93 (Local data vs. Basik’s data). These data indicate that high heterogeneity is indeed existed in the CAFs of breast cancer. The similarity was found for cytokines and chemokines genes in the CAFs. The common cytokines-CCL18 was obtained in comparing the current data with Basik’s microarray data.

### CAFs Provide a Suitable Microenvironment for Tumor Cell Invasion

**Figure 8:**
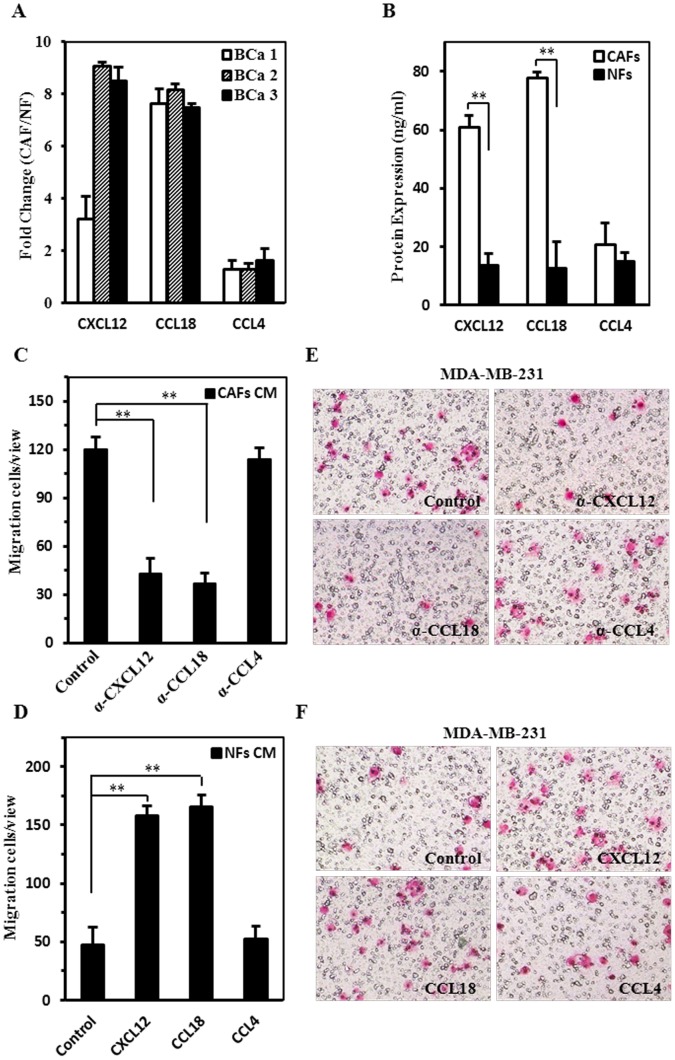
CCL18 and CXCL12 secreted by CAFs promote tumor cell invasion in cross-talk between CAFs and tumor cells. A. The mRNA levels of CXCL12, CCL18 and CCL4 were analyzed by qRT-PCR in CAF and NF from 3 patients with breast cancer. B. The protein expression levels of CXCL12, CCL18 and CCL4 were analyzed by ELISA in CAF and NF from 3 patients with breast cancer. The data were shown as fold change in CAF vs. NF. C-F. Transwell migration assays were used to determine the functional significance of cytokines secreted by CAFs on tumor invasion. Assays were conducted by using either immunoneutralizing antibody (C and E) or addition of ligands (D and F) in the co-culture system. The data were shown as mean±SD for N = 3 separate experiments (**P<0.01), (magnification 100×).

Immune-related genes, including various chemokines (CXCL12, CCL4 and CCL18), are also differentially expressed in CAFs (Fig. 6C and 8A). Chemokines secreted by stromal cells surrounding tumor tissue have been associated with tumor cell migration, invasion and angiogenesis through cross-talk between tumor and stromal cells [30]. To understand whether CXCL12, CCL4 and CCL18 involving in the cross-talk between CAFs and tumor cells, tumor cell invasion was analyzed in the co-culture system of CAFs and MDA-MB-231, or NFs and MDA-MB-231 cells. As shown in Fig. 8B and 8D, the addition of CXCL12 or CCL18 immunoneutralizing antibody in the co-culture system of CAFs and MDA-MB-231 reduced tumor cell invasion by nearly 60%. However, Condition medium (CM) with neutralizing antibody CCL4 had no significantly affect on tumor cell invasion. On the other hand, the addition of CXCL12 or CCL18 ligand, not CCL4 ligand in the co-culture system of NFs and MDA-MB-231 enhanced transmigration of tumor cells by around 3-fold (Fig. 8C and 8E). Collectively, these studies indicate that the up-regulated chemokine genes in CAFs have contributed to suitable microenvironments for tumor cell invasion.

## Discussion

Cancer-associated fibroblasts have been recognized for their impact on the carcinogenesis, promotion and progression of many carcinomas [31]. Breast cancer is notoriously associated with tumor microenvironment stroma with activated fibroblasts [32], [33]. However, relatively little is known about the relationship of biological characteristics and genetic heterogeneity between CAFs and NFs in the stroma of human breast carcinoma. Herein, this study focused on the gene expression profile of human breast cancer-derived fibroblasts in order to reveal the molecular mechanism of how fibroblasts induce a favorable microenvironment to cancer development.

CAFs had several pivotal biological characteristics compared with NFs in human breast cancer. Using the CAFs and its paired NFs isolated from breast cancer tissue, the proliferation, migration and invasion of CAFs themselves were memorably higher than that of NFs in breast cancer. And CAFs had the potential capacity for the proliferation and invasion of tumor cells. Furthermore, the invasion ability of CAFs and breast cancer cell MDA-MB-231 could significantly be increased under the co-culture system of tumor cells and CAFs or NFs cells.

In line with the finding of the biological difference between CAFs and NFs, the heterogeneity of genes expression profiles and signal pathways were highly consistent with the biological characteristics of CAFs and NFs. The huge heterogeneity of gene expression was found between CAFs and NFs (Table S1 and S2). For example, cyclin-dependent kinase 1 (CDK1), polo-like kinase 1 (PLK1), and cancer susceptibility candidate 5 (CASC5) took part in the regulating proliferation of CAF itself and tumor cell [34].

Compared with NFs, over-expressed genes in CAFs afford several interesting insights into the dynamics of the carcinoma microenvironment through the special GO Catalogue. The gene ontology and signaling pathway analysis showed that the cell cycle and cell cycle regulated signaling pathways in CAFs were strong activated for 4 and 6 times respectively, compared with NFs. Traditionally, p53, BRCA1, and BRCA2 were cancer suppressor gene, but p53 signaling pathway abnormal activated and over-expressed of BRCA1, BRCA2 and ATR in CAFs may involve in other function in the tumor microenvironment [22], [35]. Similarly, ATM signaling pathway and cdc25 and chk1 regulatory pathway may be related to cell proliferation in response to oxidative stress, although they were in responds to DNA damage or DNA strand break in traditionally [22], [23]. These data demonstrated that the vigorous proliferation of CAFs than NFs by some special activated signal pathway. However, the details will be further study for our next work.

Moreover, the different invasion capacity between CAFs and NFs may be supported by other sets of genes expression (e.g. FOXM1, MMP9, PTTG1, IGHG1, TM4SF19, S100A9, SDCBP) in the CAFs and NFs. Heterogeneity of Forkhead box M1 (FOXM1), matrix metallopeptidase 9 (MMP9), and pituitary tumor-transforming 1 (PTTG1) were involved in cancer invasion and migration [36], [37]. In breast cancer, the securin protein stability of PTTG1 was correlated with securin accumulation [38], [39]. Immunoglobulin heavy constant gamma 1 (IGHG1) and transmembrane 4 L six family member 19 (TM4SF19) were related to tumor immune invasion mechanisms and then made the tumor cells immunity [40], [41]. S100 calcium binding protein A9 (S100A9) has been revealed as the metastasis-inducing protein [42]. Syndecan binding protein (SDCBP) was reported involving in cancer metastatic progression [43], [44]. These results support the function of fibroblasts in breast carcinoma to promote cancer cell invasion, migration, and metastasis.

Furthermore, CAFs recruited numerous cells related with tumor angiogenesis, infiltration, which promoted the tumor invasion and metastasis, through secreted some special chemical chemotactic factors. For example, CXCL14 derived from CAFs in prostate cancer and SDF1/CXCL12 derived from CAFs in breast cancer called up macrophages, endothelial cells derived from bone marrow source, M2 mononuclear cells to the tumor tissue respectively, which assisted the tumor formation new blood and lymph vessels [45], [46]. Chemokine signaling pathway and PI3k signaling pathways enriched in CAFs also illustrated that CAFs could promote itself and the cancer cell invasion and migration [47], [48]. These studies suggest that a close and frequent crosstalk between the CAFs and tumor cells exists during tumor invasion and metastasis process.

In addition, a set of signaling associated with immune response, activation or immune escape were found in this study. For example, natural killer cell mediated cytotoxicity, Fc gamma R-mediated phagocytosis, antigen processing and presentation, immune network for IgA production, Lck and Fyn tyrosine kinases in initiation of TCR Activation, B lymphocyte cell surface molecules are abnormality compared with NFs. Although, its roles in the microenvironment to promote tumorigenesis and/or development for breast carcinoma are keeping study, these finding in current work indicate that CAFs may involve in immune response and/or immune escape of tumor cell.

## Supporting Information

Table S1 **Genes up-regulated in CAFs compared with NFs. (** ***Fold change ≥5*** **).**
(DOC)

Table S2 **Genes down-regulated in CAFs compared with NFs. (** ***Fold change*** **≤** ***−1.8*** **).**
(DOC)

Table S3 **Gene ontology analysis of common up-regulated genes in CAFs compared with NFs. (Only genes in the top-five ranking of each group are given).**
(DOC)
