# Supplementary material for: Biological Characteristics and Genetic Heterogeneity between Carcinoma-Associated Fibroblasts and Their Paired Normal Fibroblasts in Human Breast Cancer
Source: PLoS One. 2013 Apr 5;8(4):e60321. doi: 10.1371/journal.pone.0060321 (PMC3618271; doi:10.1371/journal.pone.0060321)
Supplement: Table S1 — Genes up-regulated in CAFs compared with NFs. ( Fold change ≥5 ). (DOC) [file pone.0060321.s001.doc]

**Table S1.** Genes up-regulated in CAFs compared with NFs. (*Fold change ≥5*)

| ***Agilent probe***  ***set*** | ***Gene symbol*** | ***Fold difference***  ***(CAF/NF)*** | ***GenBank no.*** | ***Gene name*** |
| --- | --- | --- | --- | --- |
| A_24_P319001 | GSTT1 | 57.33205 | NM_000853 | glutathione S-transferase theta 1 |
| A_23_P55270 | CCL18 | 42.05709 | NM_002988 | chemokine (C-C motif) ligand 18 (pulmonary and  activation-regulated) |
| A_24_P42264 | LYZ | 24.856487 | NM_000239 | lysozyme (renal amyloidosis) |
| A_23_P214627 | AIF1 | 22.0382 | NM_004847 | allograft inflammatory factor 1 |
| A_23_P63390 | FCGR1B | 20.276722 | NM_001017986 | Fc fragment of IgG, high affinity Ib, receptor (CD64) |
| A_23_P86283 | LAPTM5 | 19.979332 | NM_006762 | lysosomal protein transmembrane 5 |
| A_23_P137366 | C1QB | 18.385721 | NM_000491 | complement component 1, q subcomponent, B chain |
| A_23_P217269 | VSIG4 | 17.518993 | NM_007268 | V-set and immunoglobulin domain containing 4 |
| A_23_P75769 | MS4A4A | 15.966262 | NM_024021 | membrane-spanning 4-domains, subfamily A, member 4 |
| A_32_P87697 | HLA-DRA | 15.46717 | NM_019111 | major histocompatibility complex, class II, DR alpha |
| _32_P70158 | LILRB3 | 14.896184 | NM_006864 | leukocyte immunoglobulin-like receptor, subfamily B  (with TM and ITIM domains), member 3 |
| A_24_P365767 | CYBB | 14.523735 | NM_000397 | cytochrome b-245, beta polypeptide |
| A_23_P257111 | FBP1 | 14.050103 | NM_000507 | fructose-1,6-bisphosphatase 1 |
| A_23_P85800 | CD52 | 13.628998 | NM_001803 | CD52 molecule |
| A_23_P200728 | FCGR3A | 12.939728 | NM_000569 | Fc fragment of IgG, low affinity IIIa, receptor (CD16a) |
| A_23_P118834 | TOP2A | 12.872156 | NM_001067 | topoisomerase (DNA) II alpha 170kDa |
| A_23_P27994 | TYROBP | 12.298394 | NM_003332 | TYRO protein tyrosine kinase binding protein |
| A_23_P74547 | CD53 | 12.127824 | NM_001040033 | CD53 molecule |
| A_23_P74145 | CD48 | 11.9982815 | NM_001778 | CD48 molecule |
| A_24_P169873 | IGHA2 | 11.88215 | BX640625 | immunoglobulin heavy constant alpha 2 (A2m marker) |
| A_23_P88331 | DLGAP5 | 11.735786 | NM_014750 | discs, large (Drosophila) homolog-associated protein 5 |
| A_23_P372946 | TM4SF19 | 9.865864 | NM_138461 | transmembrane 4 L six family member 19 |
| A_23_P252471 | PECAM1 | 9.423389 | NM_000442 | platelet/endothelial cell adhesion molecule |
| A_23_P153562 | C5AR1 | 9.093806 | NM_001736 | complement component 5a receptor 1 |
| A_23_P106629 | IGSF6 | 8.988369 | NM_005849 | immunoglobulin superfamily, member 6 |
| A_23_P18903 | HAVCR2 | 8.903661 | NM_032782 | hepatitis A virus cellular receptor 2 |
| A_23_P259586 | TTK | 8.900507 | NM_003318 | TTK protein kinase |
| A_24_P12397 | TREM2 | 8.531248 | NM_018965 | triggering receptor expressed on myeloid cells 2 |
| A_24_P605563 | IGL | 8.48721 | BC012159 | immunoglobulin lambda locus |
| A_23_P40174 | MMP9 | 8.464997 | NM_004994 | 9 matrix metallopeptidase 9 (gelatinase B, 92kDa gelatinase, 2kDa type IV collagenase) |
| A_24_P94916 | LST1 | 8.37149 | NM_007161 | leukocyte specific transcript 1 |
| A_23_P100127 | CASC5 | 8.230998 | NM_170589 | cancer susceptibility candidate 5 |
| A_23_P121596 | PPBP | 8.100661 | NM_002704 | pro-platelet basic protein (chemokine (C-X-C motif) ligand 7) |
| A_23_P35219 | NEK2 | 7.94375 | NM_002497 | NIMA (never in mitosis gene a)-related kinase 2 |
| A_32_P62997 | PBK | 7.9390717 | NM_018492 | PDZ binding kinase |
| A_23_P209678 | PLEK | 7.872885 | NM_002664 | Pleckstrin |
| A_24_P372223 | MSR1 | 7.771098 | NM_138715 | macrophage scavenger receptor 1 |
| A_23_P9714 | RGS1 | 7.7293286 | NM_002922 | regulator of G-protein signaling 1 |
| A_24_P722155 | LOC100128098 | 7.698199 | BC029655 | hypothetical protein LOC100128098 |
| A_32_P351968 | HLA-DMB | 7.6060877 | NM_002118 | major histocompatibility complex, class II, DM beta |
| A_24_P192914 | AMICA1 | 7.590581 | NM_153206 | adhesion molecule, interacts with CXADR antigen 1 |
| A_23_P90626 | CYTIP | 7.577643 | NM_004288 | cytohesin 1 interacting protein |
| A_32_P58215 | CD84 | 7.55798 | AW451533 | CD84 molecule |
| A_23_P106362 | AQP9 | 7.458346 | NM_020980 | aquaporin 9 |
| A_23_P388812 | CKAP2L | 7.3881326 | NM_152515 | cytoskeleton associated protein 2-like |
| A_23_P150667 | KIF18A | 7.3545065 | NM_031217 | kinesin family member 18A |
| A_23_P35668 | ANLN | 6.9464073 | NM_018685 | anillin, actin binding protein |
| A_24_P222655 | C1QA | 6.76282 | NM_015991 | complement component 1, q subcomponent, A chain |
| A_32_P96719 | SHCBP1 | 6.723755 | NM_024745 | SHC SH2-domain binding protein 1 |
| A_23_P104464 | ALOX5 | 6.6286287 | NM_000698 | arachidonate 5-lipoxygenase |
| A_23_P155815 | NCAPG | 6.5944724 | NM_022346 | non-SMC condensin I complex, subunit G |
| A_23_P434809 | S100A8 | 6.5580955 | NM_002964 | S100 calcium binding protein A8 |
| A_24_P323598 | ESCO2 | 6.5055914 | NM_001017420 | establishment of cohesion 1 homolog 2 (S. cerevisiae) |
| A_24_P5103 | LOC100293440 | 6.3891716 | S76132 | similar to Ig lambda chain |
| A_23_P133956 | KIFC1 | 6.3616014 | NM_002263 | kinesin family member C1 |
| A_23_P109988 | CD86 | 6.2443314 | NM_006889 | CD86 molecule |
| A_23_P34644 | FCGR2B | 6.2036843 | NM_004001 | Fc fragment of IgG, low affinity IIb, receptor (CD32) |
| A_23_P115872 | CEP55 | 6.114935 | NM_018131 | centrosomal protein 55kDa |
| A_23_P52017 | ASPM | 6.071567 | NM_018136 | asp (abnormal spindle) homolog, microcephaly associated (Drosophila) |
| A_23_P58321 | CCNA2 | 6.033161 | NM_001237 | cyclin A2 |
| A_24_P766716 | CMKLR1 | 5.9867244 | NM_001142343 | chemokine-like receptor 1 |
| A_23_P23048 | S100A9 | 5.9266834 | NM_002965 | S100 calcium binding protein A9 |
| A_23_P31006 | HLA-DRB5 | 5.9127684 | NM_002125 | major histocompatibility complex, class II, DR beta 5 |
| A_24_P234196 | RRM2 | 5.8427906 | NM_001034 | ribonucleotide reductase M2 |
| A_24_P82749 | CD37 | 5.827317 | NM_001774 | CD37 molecule |
| A_23_P412562 | C1orf162 | 5.8058367 | NM_174896 | chromosome 1 open reading frame 162 |
| A_23_P74349 | NUF2 | 5.698047 | NM_145697 | NUF2, NDC80 kinetochore complex component, homolog (S. cerevisiae) |
| A_24_P148717 | CCR1 | 5.647212 | NM_001295 | chemokine (C-C motif) receptor 1 |
| A_23_P401 | CENPF | 5.603279 | NM_016343 | centromere protein F, 350/400ka (mitosin) |
| A_23_P163251 | PAQR5 | 5.5370083 | NM_017705 | progestin and adipoQ receptor family member V |
| A_23_P118174 | PLK1 | 5.5016675 | NM_005030 | polo-like kinase 1 (Drosophila) |
| A_23_P138194 | NCF2 | 5.490558 | NM_000433 | neutrophil cytosolic factor 2 |
| A_23_P250385 | HIST1H1B | 5.4420223 | NM_005322 | histone cluster 1, H1b |
| A_23_P46356 | TNFAIP8L2 | 5.303782 | NM_024575 | tumor necrosis factor, alpha-induced protein 8-like 2 |
| A_23_P51085 | SPC25 | 5.2511554 | NM_020675 | SPC25, NDC80 kinetochore complex component, homolog (S. cerevisiae) |
| A_24_P14156 | NDC80 | 5.2166114 | NM_006101 | NDC80 homolog, kinetochore complex component (S. cerevisiae) |
| A_24_P10214 | STXBP6 | 5.1927266 | NM_014178 | syntaxin binding protein 6 (amisyn) |
| A_23_P3014 | RNASE6 | 5.192071 | NM_005615 | ribonuclease, RNase A family, k6 |
| A_23_P155057 | CYTH4 | 5.1729307 | NM_013385 | cytohesin 4 |
| A_23_P30547 | LCP2 | 5.1694794 | NM_005565 | lymphocyte cytosolic protein 2 (SH2 domain containing  leukocyte protein of 76kDa) |
| A_23_P138507 | CDK1 | 5.0987124 | NM_001786 | cyclin-dependent kinase 1 |
| A_23_P52278 | KIF11 | 5.0651217 | NM_004523 | kinesin family member 11 |

**Note:** CAFs, carcinoma-associated fibroblasts; NFs, normal fibroblasts.
