# Supplementary material for: Biological Characteristics and Genetic Heterogeneity between Carcinoma-Associated Fibroblasts and Their Paired Normal Fibroblasts in Human Breast Cancer
Source: PLoS One. 2013 Apr 5;8(4):e60321. doi: 10.1371/journal.pone.0060321 (PMC3618271; doi:10.1371/journal.pone.0060321)
Supplement: Table S2 — Genes down-regulated in CAFs compared with NFs. ( Fold change ≤ −1.8 ). (DOC) [file pone.0060321.s002.doc]

**Table S2.** Genes down-regulated in CAFs compared with NFs. (*Fold change ≤ -1.8*)

| ***Agilent probe***  ***set*** | ***Gene symbol*** | ***Fold difference***  ***(CAF/NF)*** | ***GenBank no.*** | ***Gene name*** |
| --- | --- | --- | --- | --- |
| A_32_P78681 | GLP2R | 1.8575919 | BC043390 | glucagon-like peptide 2 receptor |
| A_23_P109171 | BFSP1 | 1.9543742 | NM_001195 | beaded filament structural protein 1, filensin |
| A_23_P209449 | FZD7 | 2.2879894 | NM_003507 | frizzled homolog 7 (Drosophila) |
| A_32_P310335 | JAM2 | 2.4655752 | AK056079 | junctional adhesion molecule 2 |
| A_23_P170110 | NA | 2.6253881 | AF227517 | NA |
| A_32_P35759 | NA | 2.8111758 | BX118285 | NA |
| A_24_P169048 | RFPL3S5 | 2.823076 | NR_001450 | RFPL3 antisense RNA (non-protein coding) |
| A_23_P40415 | ADAMTS | 3.0411544 | NM_007038 | ADAM metallopeptidase with thrombospondin type 1 motif, 5 |
| A_24_P76313 | FAM43B | 3.2783325 | NM_207334 | family with sequence similarity 43, member B |
| A_23_P13907 | IGF1 | 3.3583279 | NM_000618 | insulin-like growth factor 1 (somatomedin C) |
| A_23_P90453 | KRTDAP | 3.4243267 | NM_207392 | keratinocyte differentiation-associated protein |
| A_24_P304419 | IGF1 | 3.4966352 | NM_000618 | insulin-like growth factor 1 (somatomedin C) |
| A_32_P85732 | C9orf135 | 3.51287 | NM_001010940 | chromosome 9 open reading frame 135 |
| A_32_P205624 | SHC2 | 4.711804 | NM_012435 | SHC (Src homology 2 domain containing) transforming protein 2 |
| A_32_P219460 | NA | 5.6622376 | AK130896 | NA |

**Note:** CAF, carcinoma-associated fibroblasts; NA, not annotated; NF, normal fibroblasts.
