# Supplementary material for: Biological Characteristics and Genetic Heterogeneity between Carcinoma-Associated Fibroblasts and Their Paired Normal Fibroblasts in Human Breast Cancer
Source: PLoS One. 2013 Apr 5;8(4):e60321. doi: 10.1371/journal.pone.0060321 (PMC3618271; doi:10.1371/journal.pone.0060321)
Supplement: Table S3 — Gene ontology analysis of common up-regulated genes in CAFs compared with NFs. (Only genes in the top-five ranking of each group are given). (DOC) [file pone.0060321.s003.doc]

**Table S3.** Genes ontology analysis of common up-regulated genes in CAFs compare with NFs. (Only genes in the top-five ranking of each group are given)

| ***Genes Ontology (%)*** | ***Accession no.*** | ***Description*** | ***Ratio*** |
| --- | --- | --- | --- |
| Protein binding (38.5%) | BX640625  NM_000442  NM_003318  NM_170589  NM_002497 | immunoglobulin heavy constant alpha 2 (A2m marker)  platelet/endothelial cell adhesion molecule  TTK protein kinase  cancer susceptibility candidate 5  NIMA (never in mitosis gene a)-related kinase 2 | 11.88  9.42  8.90  8.23  7.94 |
| Cell cycle  (13.44%) | NM_018685  NM_001017420  NM_001237  NM_145697  NM_020675 | anillin, actin binding protein  establishment of cohesion 1 homolog 2 (S. cerevisiae)  cyclin A2  NUF2, NDC80 kinetochore complex component, homolog  SPC25, NDC80 kinetochore complex component, homolog | 6.95  6.50  6.03  5.70  5.25 |
| Integral to membrane (9.69%) | NM_007268  NM_138461  NM_032782  NM_007161  NM_002118 | V-set and immunoglobulin domain containing 4  transmembrane 4 L six family member 19  hepatitis A virus cellular receptor 2  leukocyte specific transcript 1 )  major histocompatibility complex, class II, DM beta | 17.52  9.87  8.90  8.37  7.61 |
| Cell division  (8.91%) | NM_022346  NM_002263  NM_018131  NM_018136  NM_001786 | non-SMC condensin I complex, subunit G  kinesin family member C1  centrosomal protein 55kDa  asp (abnormal spindle) homolog, microcephaly associated  cyclin-dependent kinase 1 | 6.59  6.36  6.11  6.07  5.10 |
| Signal transduction (6.46%) | M17380  NM_001400  NM_021983  NM_021201  NM_000867 | major histocompatibility complex, class II, DR beta 3  sphingosine-1-phosphate receptor 1  major histocompatibility complex, class II, DR beta 4  membrane-spanning 4-domains, subfamily A, member 7  5-hydroxytryptamine (serotonin) receptor 2B | 4.66  4.53  3.80  3.54  3.35 |
| Extracellular region (4.78%） | NM_000239  NM_000491  NM_004994  NM_020070  AK129787 | lysozyme (renal amyloidosis)  complement component 1, q subcomponent, B chain  matrix metallopeptidase 9 (gelatinase B, 92kDa gelatinase, 92kDa type IV collagenase)  immunoglobulin lambda-like polypeptide 1  immunoglobulin heavy constant gamma 1 (G1m marker) | 24.86  18.39  8.46  5.57  5.36 |
| Integral to plasma membrane  (4.13%) | NM_006762  NM_019111  NM_006864  NM_001803  NM_003332 | lysosomal protein transmembrane 5  major histocompatibility complex, class II, DR alpha  leukocyte immunoglobulin-like receptor, subfamily B (with TM and ITIM domains), member 3  CD52 molecule  TYRO protein tyrosine kinase binding protein | 19.98  15.47  14.90  13.63  12.30 |
| Cell proliferation (4.01%) | NM_016343  NM_005030  NM_004336  NM_020242  NM_001311 | centromere protein F, 350/400ka (mitosin)  polo-like kinase 1 (Drosophila)  budding uninhibited by benzimidazoles 1 homolog (yeast)  kinesin family member 15  cysteine-rich protein 1 (intestinal) | 5.60  5.50  4.76  4.18  3.79 |
| Transcription factor activity (3.49%) | NM_173576  NM_001031680  NM_014109  NM_202002  NM_004219 | mohawk homeobox  runt-related transcription factor 3  ATPase family, AAA domain containing 2  forkhead box M1  pituitary tumor-transforming 1 | 4.31  4.22  4.05  3.64  3.04 |
| Protein transport (3.23%） | NM_031217  NM_005733  NM_006544  NM_003262  NM_012416 | kinesin family member 18A  kinesin family member 20A  exocyst complex component 5  SEC62 homolog (S. cerevisiae)  RAN binding protein 6 | 7.35  4.53  3.19  2.32  2.15 |
| Receptor activity (2.97%) | NM_001017986  NM_000569  NM_018965  NM_001142343  NM_017705 | Fc fragment of IgG, high affinity Ib, receptor (CD64)  Fc fragment of IgG, low affinity IIIa, receptor (CD16a)  triggering receptor expressed on myeloid cells 2  chemokine-like receptor 1  progestin and adipoQ receptor family member V | 20.28  12.94  7.42  5.99  5.54 |
| Apoptosis (2.58%) | NM_001012271  NM_145018  NM_001211  NM_005225  NM_003608 | baculoviral IAP repeat-containing 5  chromosome 11 open reading frame 82  budding uninhibited by benzimidazoles 1 homolog beta (yeast)  E2F transcription factor 1  G protein-coupled receptor 65 | 4.78  4.63  3.72  3.08  2.99 |
| Cell differentiation (2.07%) | NM_001147  NM_001040152  NM_007174  NM_032281  NM_018154 | angiopoietin 2  paternally expressed 10  citron (rho-interacting, serine/threonine kinase 21)  ELAV (embryonic lethal, abnormal vision, Drosophila)-like 3 (Hu antigen C)  ASF1 anti-silencing function 1 homolog B (S. cerevisiae) | 4.73  3.57  3.22  2.80  2.21 |
| Cell adhesion  (1.81%) | NM_000442  NM_000887  NM_002984  NM_005578  NM_014442 | platelet/endothelial cell adhesion molecule  integrin, alpha X (complement component 3 receptor 4 subunit)  chemokine (C-C motif) ligand 4  LIM domain containing preferred translocation partner in lipoma  sialic acid binding Ig-like lectin 8 | 9.42  3.80  3.01  2.29  2.21 |
| Signal transducer activity (1.55%) | NM_002965  NM_000609  NM_004335  NM_018098  NM_004898 | S100 calcium binding protein A9  chemokine (C-X-C motif) ligand 12 (stromal cell-derived factor 1)  bone marrow stromal cell antigen 2  epithelial cell transforming sequence 2 oncogene  clock homolog (mouse) | 5.93  4.94  3.25  2.68  2.26 |
| Cell-cell signaling (1.42%） | NM_002988  NM_014750  NM_015991  NM_015136  NM_000459 | chemokine (C-C motif) ligand 18 (pulmonary and  activation-regulated)  discs, large (Drosophila) homolog-associated protein 5  complement component 1, q subcomponent, A chain  stabilin 1  TEK tyrosine kinase, endothelial | 42.06  11.74  6.76  3.54  2.57 |
| Cell junction  (1.03%) | NM_153206  NM_004004  NM_032387  NM_001635  AK128645 | adhesion molecule, interacts with CXADR antigen 1  gap junction protein, beta 2, 26kDa  WNK lysine deficient protein kinase 4  amphiphysin  syndecan binding protein (syntenin) | 7.59  4.53  3.47  2.71  2.32 |
